# Acute kidney injury in patients with Covid-19 in a Brazilian ICU: incidence, predictors and in-hospital mortality

**DOI:** 10.1590/2175-8239-JBN-2020-0144

**Published:** 2021-02-10

**Authors:** Rafael Lessa da Costa, Taíza Corrêa Sória, Eliene Ferreira Salles, Ana Venâncio Gerecht, Maurício Faria Corvisier, Márcia Adélia de Magalhães Menezes, Carla da Silveira Ávila, Eduardo Costa de Freitas Silva, Sara Regina Neto Pereira, Luiz Fernando Nogueira Simvoulidis

**Affiliations:** 1 Hospital Unimed-Rio, Rio de Janeiro, RJ, Brasil.; 2 Instituto Unimed-Rio, Rio de Janeiro, RJ, Brasil.

**Keywords:** Acute Kidney Injury, Coronavirus Infections, Covid-19, Betacoronavirus, SARS-CoV-2, Intensive Care Units, Mortality, Lesão Renal Aguda, Infecções por Coronavirus, Covid-19, Betacoronavirus, Sars-CoV-2, Unidades de Terapia Intensiva, Mortalidade

## Abstract

**Introduction:**

There is little data in the literature on acute kidney injury (AKI) in Covid-19 cases, although relevant in clinical practice in the ICU, especially in Brazil. Our goal was to identify the incidence of AKI, predictive factors and impact on hospital mortality.

**Method:**

Retrospective cohort of patients with Covid-19 admitted to the ICU. AKI was defined according to KDIGO criteria. Data was collected from electronic medical records between March 17 and April 26.

**Results:**

Of the 102 patients, 55.9% progressed with AKI, and the majority (66.7%) was classified as stage 3. Multivariate logistic regression showed age (RC 1.101; 95% CI 1.026 - 1.181; *p* = 0.0070), estimated glomerular filtration rate - eGFR (RC 1.127; 95% CI 1.022 - 1.243; *p* = 0.0170) and hypertension (RC 3.212; 95% CI 1.065 - 9.690; *p* = 0.0380) as independent predictors of AKI. Twenty-three patients died. In the group without kidney injury, there were 8.9% deaths, while in the group with AKI, 33.3% of patients died (RR 5.125; 95% CI 1.598 - 16.431; *p* = 0.0060). The average survival, in days, was higher in the group without AKI. Cox multivariate analysis showed age (RR 1.054; 95% CI 1.014 - 1.095; *p* = 0.0080) and severe acute respiratory distress syndrome (RR 8.953; 95% CI 1.128 - 71.048; p = 0.0380) as predictors of hospital mortality.

**Conclusion:**

We found a high incidence of AKI; and as predictive factors for its occurrence: age, eGFR and hypertension. AKI was associated with higher hospital mortality.

## Resumo

**Introdução:**

A lesão renal aguda (LRA)na Covid-19, apesar de relevante na prática clínica em UTI, dispõe de poucos dados na literatura, sobretudo no Brasil. Objetivo foi identificar a incidência de LRA, fatores preditores e impacto na mortalidade hospitalar.

**Método:**

Coorte retrospectiva de pacientes com Covid-19 internados em UTI. LRA foi definida segundo critérios de KDIGO. Dados coletados de registros de prontuários eletrônicos entre 17 de março e 26 de abril.

**Resultados:**

Dos 102 pacientes, 55,9% evoluíram com LRA e a maioria (66,7%) foi classificada como estágio 3. Regressão logística multivariada mostrou idade (RC 1,101; IC 95% 1,026 - 1,181; *p* = 0,0070), taxa de filtração glomerular estimada - TFGe (RC 1,127; IC 95% 1,022 - 1,243; *p* = 0,0170) e hipertensão (RC 3,212; IC 95% 1,065 - 9,690; *p* = 0,0380) como preditores independentes de LRA. Vinte e três pacientes faleceram. No grupo sem lesão renal ocorreu 8,9% de óbitos, enquanto que no grupo com LRA 33,3% dos pacientes morreram (RR 5,125; IC 95% 1,598 - 16,431; *p* = 0,0060). A média de sobrevida, em dias, foi maior no grupo sem LRA. Análise multivariada de Cox mostrou idade (RR 1,054; IC 95% 1,014 - 1,095; *p* = 0,0080) e síndrome do desconforto respiratório agudo grave (RR 8,953; IC 95% 1,128 - 71,048; *p* = 0,0380) como preditores de mortalidade hospitalar.

**Conclusão:**

Encontramos alta incidência de LRA; e como fatores preditores para sua ocorrência: idade, TFGe e hipertensão. A LRA estava associada a maior mortalidade hospitalar.

## Introduction

In December 2019, there were a series of pneumonia cases in Wuhan, Hubei province, China. Quickly, thousands of patients evolved with the same condition and, subsequently, the causal agent was identified: severe acute respiratory syndrome coronavirus 2 (Sars-CoV-2)^1^. In March, the World Health Organization (WHO) declared the disease caused by the new coronavirus (Covid-19) a global health problem^2^. Currently, there are more than 29 million people infected in the world, with at least 925 thousand dead, and Brazil is the third most affected country by the disease^3^.

Among the organic dysfunctions related to Covid-19, hypoxemic respiratory failure received greater prominence^4^, but acute kidney injury (AKI) has also been reported. A cohort in China, with 1,099 patients reported 5% of admissions to the Intensive Care Unit (ICU), 3.4% of Acute Respiratory Distress Syndrome (ARDS) and 0.5% of AKI in the general sample. Among the individuals considered with severe Covid-19 the incidences of ARDS and AKI were 15.6% and 2.9%, respectively^5^. Another population, also in China, with 111 patients without previous kidney disease did not present any case of AKI^6^. Nonetheless, a meta-analysis with a predominance of eastern population showed that, despite the incidence of only 3% of AKI among hospitalized patients, this number reached 19% when considering patients admitted to the ICU^7^. Some authors recommend attention concerning the emergence of renal dysfunction in patients infected with the new coronavirus, as there is an increase in morbidity, and there is still no specific treatment for either the viral infection or the AKI it causes^8^^,^ 9.

In Brazil, data on the association between AKI and Covid-19 are still incipient. Understanding its behavior in these patients can be relevant for therapeutic optimization, supply logistics and improvement of clinical outcomes.

The primary objective of this study is to investigate the incidence of AKI and the possible predictive factors for its occurrence in patients admitted with Covid-19 in the ICU of a private hospital in Rio de Janeiro, Brazil; and as a secondary objective, assess its impact on in-hospital mortality.

## Methods

### Study design

This is a retrospective cohort study carried out in a private hospital in the city of Rio de Janeiro, Brazil, by consulting the electronic medical record system of patients consecutively admitted to the ICU with a diagnosis of Covid-19, confirmed by polymerase chain reaction from an oropharyngeal swab, according to WHO criteria^10^. This study ran from March 17 to April 26.

### Population

Patients were classified according to their AKI stages. The patients with chronic kidney disease and an estimated glomerular filtration rate (eGFR) of less than 30 mL/min/1.73m², or who underwent renal replacement therapy by any method prior to admission, were excluded. All individuals were over 18 years old.

### Definition of acute kidney injury

For the AKI diagnosis and stratification, we used the Kidney Disease Improving Global Outcomes (KDIGO) criteria: stage 1 - increase in serum creatinine from 0.3 mg/dL in 48 hours or increase from 1.5 to 1.9 value of baseline serum creatinine within 7 days; stage 2 - 2 to 2.9-fold increase in serum creatinine within 7 days or urine output below 0.5 mL/kg/h for more than 12 hours; and stage 3 - 3-fold increase in serum creatinine in 7 days or creatinine higher than 4 mg/dL or initiation of renal replacement therapy through hemodialysis or urine output below 0.3 mL/kg/h for 24 hours or more, or anuria for 12 hours or more^11^. The value of creatinine used as baseline was measured upon admission to the ICU.

### Sample characteristics

We used the following info to describe the characteristics of the population: age, sex, body mass index - BMI (Kg/m^2^), systemic arterial hypertension (SAH), diabetes mellitus (DM), lung disease (asthma and lung disease) chronic obstructive pulmonary disease - COPD), cardiovascular disease (known coronary artery disease or any degree of left ventricular dysfunction), solid organ neoplasia, date on symptom onset, length of ICU stay, length of hospital stay and most common clinical complications (acute respiratory distress syndrome - according to the Berlin definitions^12^, need for invasive ventilatory support, use of vasopressor drugs, venous thromboembolism and death).

Data collection was performed by AVG and MFC - medical researchers. In cases of doubt or divergence of records, medical researcher RLC was responsible for the final decision.

### Ethical aspects

The Research Ethics Committee of the State University of Rio de Janeiro (UERJ) under number 4,036,509 approved this study. The free and informed consent form was waived because this was a retrospective cohort study.

### Statistical analysis

We expressed the continuous variables as means, standard deviations, medians and interquartiles; and the categorical variables in absolute and relative frequency. We used the Shapiro-Wilk normality test to assess continuous variables distribution. We compared the continuous variables using the Student t-test or the Mann-Whitney U-test. We compared the categorical variables using the chi-square or the Fisher's exact tests. We ran the logistic regression analysis to determine the acute kidney injury predictors. The variables associated with acute kidney injury at a significance level of *p* < 0.20 were included in the multivariate regression model. We used the stepwise forward method. We calculated the survival functions using the Kaplan-Meier non-parametric estimator. The patients were stratified by the stage of acute kidney injury. We used the log-rank test to compare the survival functions for each covariate. The risk ratios (RR) were calculated for the prognosis of variables associated with the outcomes, with 95% confidence intervals (95% CI), according to the Cox's proportional model. Initially, we ran the Cox's bivariate analysis followed by a multivariate analysis for the factors with a probable role in the outcome (*p* < 0.10). The proportionality of Cox models was verified by the Schoenfeld residual diagnostic test. The tests were two-tailed and the statistical significance was expressed as *p* < 0.05. The analyzes were performed with the SPSS 22.0.

## Results

**Figure 1:**
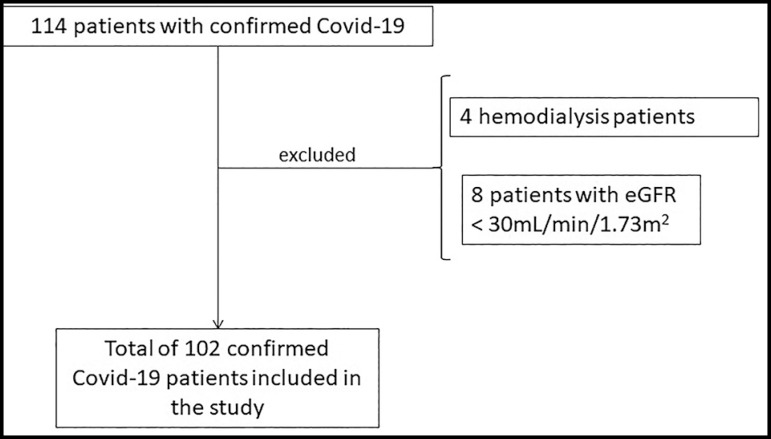
Flowchart of patients.

In the period considered by the study, a total of 114 patients were diagnosed with Covid-19 in the ICU. After applying the exclusion criteria (Figure 1), 102 patients were included in the statistical analysis.

### General characteristics and stratification by AKI

Of the 102 patients, the majority were males (58.8%), with a mean age of 66.5 years. SAH and DM were the most prevalent diseases found in this cohort, 53.9% and 31.4% of the general population, respectively. The average BMI was 28 kg/m^2^. More than a quarter of the population required dialysis, and 49% used invasive mechanical ventilation (Table 1). After stratifying the sample according to the AKI, we found a predominance of males in the group with the reported kidney injury (46.7% x 68.4%; p <0.05). The mean age in the group without AKI was 65.3 years, with no significant difference compared to the group with AKI (67.4 years). The length of hospital and ICU stay, in days, was longer in the groups with AKI (10.0 x 17.0; *p* < 0.001 and 3.0 x 15.0; *p* < 0.001, respectively). There was a predominance of hypertensive patients in the group with AKI (37.8% x 66.7%; *p* < 0.005). Moderate and severe ARDS, the need for invasive mechanical ventilation and hemodynamic support with vasopressor, were also more prevalent in the group with AKI, all with a significant p-value (Table 2).

**Table 1:** General characteristics of the 102 Covid-19 patients admitted to the ICU

Variables	General Population (n = 102)
Age	66.5 ± 15.7
Males	60 (58.8%)
eGFR (mL/min/1.73m^2^)	77.0 ± 22.6
Creatinine (mg/dL)	0.9 [0.7 - 1.1]
BMI (kg/m^2^)	28 [25 - 33]
Δ symptoms- hospitalization (days)	7 [4 - 9]
Comorbidities	
SAH	55 (53.9%)
Diabetes	32 (31.4%)
Respiratory disease^§^	8 (7.8%)
Cardiovascular disease^¶^	8 (7,8%)
Neoplasia	6 (5.9%)
Complications	
Hemodialysis	27 (26.5%)
Mild ARDS	11 (10.8%)
Moderate ARDS	23 (22.5%)
Severe ARDS	23 (22.5%)
Mechanical ventilation	50 (49.0%)
Vasopressor	48 (47.0%)
VTE	16 (15.7%)
Time in ICU	8.5 [3.0 - 17.5]
Hospital stay duration (days)	14 [8.0 - 19.2]
Death	23 (22.5%)

§: asthma and chronic obstructive pulmonary disease; coronary disease and ventricular dysfunction; Δ symptoms-hospitalization: time from symptom onset to the moment of hospitalization; SAH: systemic arterial hypertension; BMI: body mass index; ARDS: acute respiratory distress syndrome; VTE: venous thromboembolism.

**Table 2:** General characteristics of the 102 patients with Covid-19 admitted to the ICU, stratified by the acute kidney injury

Variable	Without AKI (n = 45)	AKI (n = 57)	Stages of AKI			p-value
			Stage 1 (n = 10)	Stage 2 (n = 9)	Stage 3 (n = 38)	
**Characteristics**						
**Age**	65.3±15.9	67.4±15.6	60.5 [53.4 - 87.3]	69.1 [54.0 - 74.1]	71.4 [56.9 - 82.0]	0.9220
**Male gender**	21 (46.7%)	39 (68.4%)	6 (60%)	7 (77.8%)	26 (68.4%)	**0.0420**
**eGRF** **(mL/** **min/1.73m^2^)**	79.2±18.9	75.3±25.1	90.0 [65.8 - 105]	83.8 [78.7 - 95.3]	67,0 [51.2 - 85.8]	0.0710
**Creatinine** **(mg/dL)**	0.9 [0.7-1.0]	1.0 [0.8 - 1.2]	0.8 [0.6 - 1.0]	0.8 [ 0.7 - 1.0]	1.1 [0.9 - 1.3]	**0.049**
**BMI (kg/m^2^)**	27.0 [23.4-31.7]	29.9 [27.0-34.6]	28.9 [24.2-33.0]	28.0 [26.7-37.1]	30.5 [27.3 - 35.0]	**0.0180**
**Δ symptoms-** **hospitalization** **(days)**	8.0 [4.7 - 10.0]	6 [3.8 - 8.2]	7.5 [2.0 - 10.0]	7.0 [3.8 - 9.5]	5.5 [4.0 - 7.0]	0.2070
**Comorbidities**						
**SAH**	17 (37.8%)	38 (66.7%)	4 (40%)	5 (55.6%)	29 (76.3%)	**0.0050**
**Diabetes**	15 (33.3%)	17 (29.8%)	3 (30%)	4 (44.4%)	10 (26.3%)	0.08300
**Respiratory** **diseases^§^**	2 (4.4%)	6 (10.5%)	1 (10%)	0 (0%)	5 (13.2%)	0.4610
**Cardiovascular** **diseases^¶^**	3 (6.7%)	5 (8.7%)	1 (10%)	1 (11.1%)	3 (7.9%)	1.0000
**Neoplasia**	3 (6.7%)	3 (5.3%)	1 (10%)	1 (11.1%)	1 (2.6%)	1.0000
**Complications**						
**Hemodialysis**	0 (0%)	27(47.4%)	0 (0%)	0 (0%)	27 (71%)	**0.0001**
**Mild ARDS**	5 (11.1%)	6 (10.5%)	0 (0%)	2 (22.2%)	4 (10.5%)	1.0000
**Moderate ARDS**	5 (11.1%)	18 (31.6%)	2 (20%)	2 (22.2%)	14 (36.8%)	**0.0170**
**Severe ARDS**	2 (4.4%)	21 (36.8%)	1 (10%)	2 (22.2%)	18 (47.4%)	**0.0001**
**Mechanical ventilation**	8 (17.8%)	42 (73.7%)	2 (20%)	5 (55.6%)	35 (92.1%)	**0.0001**
**Vasopressor**	7 (15.6%)	41 (71.9%)	2 (20%)	4 (44.4%)	35 (92.1%)	**0.0001**
**VTE**	4 (8.9%)	12 (21.1%)	2 (20%)	2 (22.2%)	8 (21.1%)	0.1080
**Time in ICU**	3.0 [2.0 - 7.3]	15 [8 - 26.5]	5.5 [4.0 - 12.0]	19.0 [9.0 - 27.5]	18.0 [11.0 - 31.0]	**0.0001**
**Hospital stay** **duration (days)**	10.0 [6.0 - 15.0]	17.0 [10.8 - 35.3]	9.5 [9.0 - 15.0]	19.0 [13.3 - 41.7]	19.5 [13.0 - 39.0]	**0.0001**
**Death**	4 (8.9%)	19 (33.3%)	0 (0%)	0 (0%)	19 (50%)	**0.0040**

§:asthma and chronic obstructive pulmonary disease; coronary disease and ventricular dysfunction; Δ symptoms-hospitalization: time since symptom onset until the time of hospitalization; SAH: systemic arterial hypertension; BMI: body mass index; ARDS: acute respiratory distress syndrome; VTE: venous thromboembolism.

### Acute kidney injury

**Figure 2:**
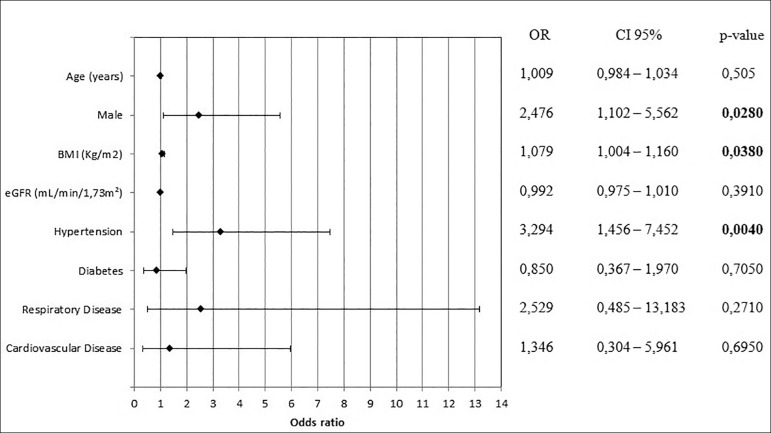
Univariate logistic regression of risk factors associated with acute kidney injury.

**Figure 3:**
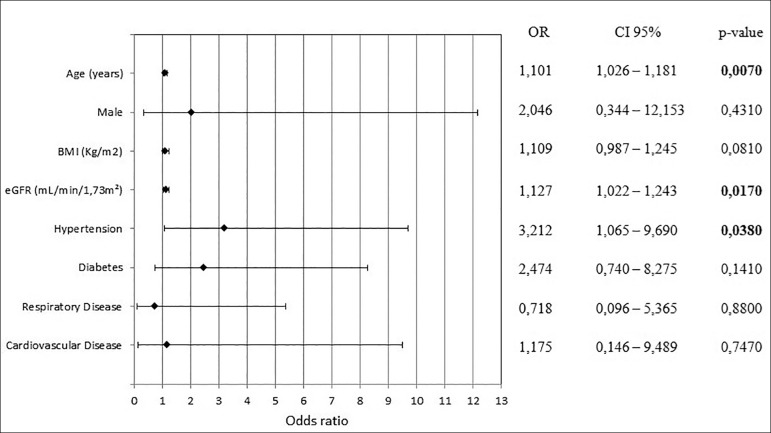
Multivariate logistic regression of risk factors associated with acute kidney injury.

In our sample, 57 patients (55.9%) evolved with some degree of AKI, so that the majority (66.7%) was classified as stage 3 (Table 2). The incidence of AKI was higher in those hospitalized with higher baseline creatinine values (0.9 mg/dL x 1.0 mg/dL, *p* < 0.05). The univariate logistic regression of previously known factors showed the following factors as predictors of acute changes in renal function (Figure 2): male (odds ratio - OR: 2.476; 95% CI 1.102 - 5.562; *p* = 0.0280), BMI (OR 1.079; 95% CI 1.004 - 1.160; *p* = 0.0380) and SAH (OR 3.294; 95% CI 1.456 - 7.452; *p* = 0.0040). However, multivariate logistic regression showed age (OR 1.101; 95% CI 1.026 - 1.181; *p* = 0.0070), eGFR (OR 1.127; 95% CI 1.022 - 1.243; *p* = 0.0170) and SAH (OR 3.212; 95% CI 1.065 - 9.690; *p* = 0.0380) as predictors of acute kidney injury (Figure 3).

### Hospital mortality

Of the total participants, 23 (22.5%) patients died. In the group of patients without kidney injury, there were 4 (8.9%) deaths, while in the group with AKI 19 (33.3%) died (HR 5,125; 95% CI 1,598 - 16,431; *p* = 0,0060). The mean survival in days was higher in the group without AKI (42.1 days; 95% CI 27.2 - 56.1) when compared to the group with acute stage 3 kidney injury (41.6 days; 95% CI 32, 1 - 52.1). In the AKI stage 1 and 2 groups, there was no death. The Log-rank test for the comparison of survival curves between the groups had a p value = 0.0136. Cox's bivariate survival analysis for mortality showed a relationship with the following factors: age (HR 1.042; 95% CI 1.010 - 1.076; *p* = 0.01), eGFR (HR 0.970; 95% CI 0.951 - 0.990; *p* = 0.004), mechanical ventilation (HR 1,852; 95% CI 1,434 - 2,392; *p* = 0.0001), use of vasopressors (HR 1.920; 95% CI 1.464 - 2.519; *p* = 0.0001), moderate ARDS (HR 2.985; CI 95% 1.079 - 8.254; *p* = 0.035) and severe ARDS (HR 8.970; 95% CI 3.144 - 25.840; *p* = 0.0001). In the Cox's multivariate analysis, only age (HR 1.054; 95% CI 1.014 - 1.095; *p* = 0.008) and severe ARDS (HR 8.953; 95% CI 1.128 - 71.048; *p* = 0.038) remained as factors associated with mortality (Table 3).

**Table 3:** Cox multivariate and bivariate survival analysis in 102 patients with acute kidney injury and Covid-19 admitted to the ICU

	Bivariate			Multivariate		
Variables	RR	CI 95%	p-value	RR	CI 95%	p-value
**Age**	1.042	1.010 - 1.076	**0.0100**	1.054	1.014 - 1.095	**0.0080**
**Males**	1.590	0.692 - 3.652	0.2750	-	-	-
**BMI (kg/m^2^)**	0.987	0.935 - 1.053	0.6930	-	-	-
**eGFR**	0.970	0.951 - 0.990	**0.0040**	0.981	0.961 - 1.001	0.0650
**Hypertension**	0.471	0.174 - 1.276	0.1390	-	-	-
**Diabetes**	0.983	0.403 - 2.398	0.9710	-	-	-
**Mechanical** **ventilation**	1.852	1.434 - 2.392	**0.0001**	1.304	0.100 - 1.148	0.9150
**Vasopressor**	1.920	1.464 - 2.519	**0.0001**	1.383	0.109 - 1.297	0.9040
**Moderate** **ARDS**	2.985	1.079 - 8.254	**0.0350**	1.387	0.049 - 3.069	0.3690
**Severe ARDS**	8.970	3.114 - 25.840	**0.0001**	8.953	1.128 - 71.048	**0.0380**

BMI: body mass index; RR: relative risk; ARDS acute respiratory distress syndrome; eGFR: estimated glomerular filtration rate.

## Discussion

Covid-19 presents clinical spectra ranging from asymptomatic patients to cases with multiple organ dysfunction and death. The AKI pathophysiology in patients with Covid-19 is still unclear, but it appears to be complex and multifactorial. It is believed that, in addition to factors such as previous comorbidity, lesions secondary to hemodynamic changes and the release of cytokines - similar to that seen in sepsis, the state of hypercoagulability and direct cytotoxicity by the virus with activation of angiotensin II are added, whereas the angiotensin II-converting enzyme is the Sars-CoV-2 receptor is highly expressed in kidney cells, as well as in lung cells^13^. In spite of having kidney dysfunction on the front lines of Covid-19, there are still few studies that aim to analyze this achievement so far.

In our sample of 102 patients with Covid-19 admitted to an ICU in Rio de Janeiro, we found an AKI incidence in 55.9% of the general population; and of the 38 patients in stage 3, more than 70% required renal replacement therapy through hemodialysis. Chinese researchers compared the incidence of renal dysfunction between three countries on different continents: China, Italy and the United States. The incidences of AKI in patients with Covid-19 in China were 0.5%, 0.1% and 2.9%, of the total of patients with mild, moderate and severe manifestations of Covid-19, respectively. Among Americans, AKI was reported in 22.2% of those hospitalized and in 72.1% of those who died. In Italy, the AKI was second only to ARDS^14^.

A study involving 701 consecutive patients admitted to a hospital in China found AKI in only 5.1% of cases, and only 2% (14/701) in stage 3. The overall mortality in this population was 16.1%, but there were higher number of deaths in the group with increased baseline creatinine (13.2% x 33.7%; *p* < 0.001). Age over 65 years, male gender and severe manifestation of Covid-19 were associated with higher hospital mortality^15^.

In the United States, researchers found AKI in 37% of the 5,449 patients admitted to 13 hospitals, and 619 (31%) in stage 3. Dialysis support was indicated for 5.2% of all cases, which corresponds to 14.3 % of those with AKI. The need for invasive ventilatory support also drew attention in this study, with more than 50% of ventilated patients in the AKI group, whereas in the other group, this number was only 3.5%. The overall mortality in the sample was 16.3%, and among those who developed renal dysfunction, 35% died. Among the independent predictive factors for renal dysfunction, we also found age, hypertension and diabetes^16^. In our sample, as independent factors for the occurrence of AKI, obtained by logistic regression; we identified systemic arterial hypertension, age and eGFR.

A retrospective study evaluated clinical and laboratory records of 333 hospitalized patients, and found criteria for AKI in 4.7% of the participants, and this number was higher among critically ill patients (42.9%). The authors also reported that only 1.2% of the patients without renal impairment during hospitalization died, while in the group with renal impairment this number was ten times higher^17^.

A systematic review involving 3,027 individuals in 13 papers analyzed the clinical characteristics of a group of critically ill and non-critically ill Covid-19 patients; those with a serum creatinine value higher than or equal to 133 mmol/L (1.5 mg/dL) were five times more likely to belong to the critically ill group^18^. Another meta-analysis and systematic review evaluated the survival of 1,277 individuals with Covid-19 who developed stage 3 AKI. The analysis showed that severe AKI is associated with a higher mortality (HR = 4.19; 95% CI 3.31 - 5.31%)^19^.

Through a cohort with 1,603 inpatients, Spanish researchers reported an incidence of 11.4% AKI, of which only 5.1% required hemodialysis. Hospital mortality was 12.3%, but it was higher among patients admitted with increased serum creatinine levels (32.4%), with chronic kidney disease (41.1%) and in those with AKI (15.9%), compared with those with normal serum creatinine (5.8%). A multivariate analysis showed an association between age and higher hospital mortality, as in our sample; and in a univariate analysis, AKI also showed a positive association, with the same outcome^20^.

The results of a cohort of 100 patients, also admitted to the ICU, were similar to what we found in our population. Most were male; hypertension and diabetes were the most prevalent diseases. AKI had an incidence of 81%, higher than that found in our sample. Multivariate analysis showed only the SOFA score as a factor associated with AKI. More than half of the patients with stage 2 and 3 AKI died before the established 28-day period, and AKI severity was associated with mortality, as well as older age and higher SOFA score^21^. A smaller cohort, with 71 patients followed for 2 weeks in the ICU, found AKI in 69% of patients during the period studied, and added to patients who have already arrived with AKI, the total number of cases reaches 80%, too. AKI stages 1, 2 and 3 showed prevalences of 35%, 35% and 30%, respectively. The study did not assess factors associated with the occurrence of AKI, nor mortality^22^.

Respiratory complications are the most frequent and worrying symptoms in patients with Covid-19. Of the patients with AKI in our cohort, 73.7% required invasive ventilatory support, and among those in AKI stage 3, more than 90% had the same need. Moderate and severe ARDS was also more prevalent in patients with acute impairment of renal function, with 31.6% and 36.8%, respectively. This data was very similar to that found in the cohort of American patients^16^. This can be explained by the great renal sensitivity to changes in blood oxygen tension. The complex inflammatory response generated by ARDS, the hemodynamic changes involved in the treatment of these patients and the acute changes in oxygenation trigger the release of inflammatory mediators that can affect renal vascular tone and the viability of renal cells, thus culminating in acute renal failure^23^. Severely ill individuals with ARDS may have higher rates of AKI, and once present, it increases the mortality rate^24^.

A cohort of 370 North American patients, consecutively hospitalized, evaluated the incidence of acute kidney injury and its effect on the mortality of patients with Covid-19. With an incidence of 54.7%, AKI, along with age and ARDS, contributed to higher hospital mortality^25^.

In our population, we found a general mortality higher than that reported in other studies that specifically assessed AKI in patients with Covid-19^15^^,^ 16 ^,^ 20; however, the highest proportion of deaths in the group with impaired renal function was similar and with a significant association. Age and severe ARDS were independent factors associated with mortality during hospitalization, according to Cox's multivariate model.

Studies in patients with Covid-19 admitted to the ICU who did not specifically consider renal involvement, reported AKI rates and deaths with wide variation. In Brescia, Italy, of the 33 patients studied, 3% required renal replacement therapy, and only 1 patient died^26^. Another Italian cohort found 26% mortality in 1,591 cases, and did not mention AKI^27^. In China, of the 52 patients studied , 29% had some acute renal function damage, 17% underwent hemodialysis and 61.5% died^28^. In the United States, mortality ranged from 50%^29^ to 52.4%^30^, and the incidence of AKI was 14.3%^30^. In Spain, AKI has not been studied, and mortality was 15% at the end of 28 days^31^.

The studies reported here were mostly retrospective cohorts; however, the different inclusion criteria, clinical presentation of Covid-19, sample size and objectives made it difficult to compare data and information.

As limitations, we have an observational study, dependent on medical records and composed of a convenience sample from a relatively small number of patients compared to cohorts that specifically investigated AKI in patients with Covid-19. As we only include patients admitted to the ICU, we believe it is one of the reasons for the number of participants found during the evaluation. We did not evaluate the temporal relationship between the moment of respiratory failure and the need for invasive mechanical ventilation with the onset of worsening renal function. As well as the proportion of individuals who had their kidney function fully recovered during the observed period was not described. The creatinine value taken as baseline was the first serum measurement upon admission to the ICU.

However, we did not find in the national literature, until the final preparation of this study, any other study on AKI in patients with Covid-19 admitted to the ICU. Certainly, our data will aggregate future research on the topic, so that they can corroborate the results presented here.

## Conclusion

We found a high incidence of AKI in our sample, and as independent predictors of its occurrence, we have age, eGFR and SAH. AKI was associated with higher in-hospital mortality, and individuals without impaired renal function or with AKI stages 1 and 2 had higher in-hospital survival compared to those in stage 3. The independent mortality predictors during hospitalization were age and severe ARDS.

## References

[1] Fan Wu, Zhao S, Yu B, Chen YM, Wang W, Song ZG (2020). A new coronavirus associated with human respiratory disease in
China. Nature.

[2] World Health Organization (2020). Coronavirus disease (Covid-19) weekly epidemiological update and weekly
operational update.

[3] Johns Hopkins University & Medicine (2020). Coronavirus resource center.

[4] Berlin DA, Gulick RM, Martinez FJ (2020). Severe Covid-19. N Engl J Med.

[5] Guan W, Ni Z, Hu Y, Liang W, Ou C, He J (2020). Clinical characteristics of coronavirus disease 2019 in
China. N Engl J Med.

[6] Wang L, Li X, Chen H, Yan S, Li D, Li Y (2020). Coronavirus disease 19 infection does not result in acute kidney
injury: an analysis of 116 hospitalized patients from Wuhan,
China. Am J Nephrol.

[7] Ng J, Luo Y, Phua K, Choonga AMTL (2020). Acute kidney injury in hospitalized patients with coronavirus
disease 2019 (Covid-19): a meta-analysis. J Infect.

[8] Ostermann M, Lumlertgul N, Forni LG, Hoste E (2020). What every intensivist should know about Covid-19 associated
acute kidney injury. J Crit Care.

[9] Gabarre P, Dumas G, Dupont T, Darmon M, Azoulay E, Zafrani L (2020). Acute kidney injury in critically ill patients with
Covid-19. Intensive Care Med.

[10] World Health Organization (2020). Clinical management of severe acute respiratory infection (SARI) when
Covid-19 disease is suspected.

[11] Kidney Disease - Improving Global Outcomes (2012). Acute Kidney Injury Work Group KDIGO clinical practice guideline
for acute kidney injury. Kidney Int Suppl.

[12] Ranieri VM, Rubenfeld GD, Thompson BT, Ferguson ND, Caldwell E, Fan E (2012). Acute respiratory distress syndrome: the Berlin
definition. JAMA.

[13] Batlle D, Soler MJ, Sparks MA, Hiremath S, South AM, Welling PA (2020). Acute kidney injury in Covid-19: emerging evidence of a distinct
pathophysiology. J Am Soc Nephrol.

[14] Chen L, Guo C (2020). Focus on kidney disease among the coronavirus disease 2019
patients: a comparative perspective between China, Italy and the United
States. Int J Clin Pract.

[15] Cheng Y, Luo R, Wang K, Zhang M, Wang Z, Dong L (2020). Kidney disease is associated with in-hospital death of patients
with Covid-19. Kidney Int.

[16] Hirsch JS, Ng JH, Ross DW, Sharma P, Shah HH, Barnett RL (2020). Acute kidney injury in patients hospitalized with
Covid-19. Kidney Int.

[17] Pei G, Zhang Z, Peng J, Liu L, Zhang C, Yu C (2020). Renal involvement and early prognosis in patients with Covid-19
pneumonia. J Am Soc Nephrol.

[18] Zheng Z, Peng F, Xu B, Zhao J, Liu H, Peng J (2020). Risk factors of critical & mortal Covid-19 cases: a
systematic literature review and meta-analysis. J Infect.

[19] Ali H, Daoud A, Mohamed MM, Salim SA, Yessayan L, Baharani J (2020). Survival rate in acute kidney injury superimposed Covid-19
patients: a systematic review and meta-analysis. Ren Fail.

[20] Portolés J, Marques M, Lopez-Sanchez P, Valdenebro M, Muñez E, Serrano M (2020). Chronic kidney disease and acute kidney injury in the Covid-19
Spanish Outbreak. Nephrol Dial Transplant.

[21] Joseph A, Zafrani L, Mabrouki A, Azoulay E, Darmon M Acute kidney injury in patients with SARS-CoV-2
infection. Ann Intensive Care.

[22] Rubin S, Orieux A, Prevel R, Garric A, Bats ML, Dabernat S (2020). Characterization of acute kidney injury in critically ill
patients with severe coronavirus disease 2019. Clin Kidney J.

[23] Basu RK, Wheeler DS (2013). Kidney-lung cross-talk and acute kidney injury. Pediatr Nephrol.

[24] Lombardi R, Nin N, Lorente JA, Frutos-Vivar F, Ferguson ND, Hurtado J (2011). An assessment of the acute kidney injury network creatinine-based
criteria in patients submitted to mechanical ventilation. Clin J Am Soc Nephrol.

[25] Nimkar A, Naaraayan A, Hasan A, Pant S, Durdevic M, Suarez C (2020). Incidence and risk factors for acute kidney injury and its effect
on mortality in patients hospitalized from Covid-19. Mayo Clin Proc Innov Qual Outcomes.

[26] Piva S, Filippini M, Turla F, Cattaneo S, Margola A, Fulviis S (2020). Clinical presentation and initial management critically ill
patients with severe acute respiratory syndrome coronavirus 2 (SARS-CoV-2)
infection in Brescia, Italy. J Crit Care.

[27] Grasselli G, Zangrillo A, Zanella A, Antonelli M, Cabrini L, Castelli A (2020). Baseline characteristics and outcomes of 1591 patients infected
with SARS-CoV-2 admitted to ICUs of the Lombardy Region,
Italy. JAMA.

[28] Yang X, Yu Y, Xu J, Shu H, Xia J, Liu H (2020). Clinical course and outcomes of critically ill patients with
SARS-CoV-2 pneumonia in Wuhan, China: a single-centered, retrospective,
observational study. Lancet Respir Med.

[29] Bhatraju PK, Ghassemieh BJ, Nichols M, Kim R, Jerome KR, Nalla AK (2020). Covid-19 in critically ill patients in the Seattle region - case
series. N Engl J Med.

[30] Arentz M, Yim E, Klaff L, Lokhandwala S, Riedo FX, Chong M (2020). Characteristics and outcomes of 21 critically ill patients with
Covid-19 in Washington State. JAMA.

[31] Barrasa H, Rello J, Tejada S, Martín A, Balziskueta G, Vinuesa C (2020). SARS-CoV-2 in Spanish intensive care early experience with 15-day
survival in Vitoria. Anaesth Crit Care Pain Med.

